# Does dihydromyricetin protect the kidney following ischemia–reperfusion injury in male rats?

**DOI:** 10.14814/phy2.70394

**Published:** 2025-06-11

**Authors:** Fayez T. Hammad, Loay Lubbad, Mariam Aljawder, Azza Al Ali, Suhail Al‐Salam, Awwab F. Hammad, Mustafa M. Ardah, M. Emdadul Haque, Jasmine Abdul Rasheed, Waheed F. Hammad

**Affiliations:** ^1^ Department of Surgery College of Medicine & Health Sciences, United Arab Emirates University Al Ain United Arab Emirates; ^2^ Department of Pathology College of Medicine & Health Sciences, United Arab Emirates University Al Ain United Arab Emirates; ^3^ School of Medicine, University of Jordan Amman Jordan; ^4^ Department of Biochemistry and Molecular Biology College of Medicine & Health Sciences, United Arab Emirates University Al Ain United Arab Emirates; ^5^ Al‐Ahliyya Amman University Amman Jordan

**Keywords:** dihydromyricetin, ischemia reperfusion injury, renal

## Abstract

Efforts to prevent the deleterious effects of the ischemia–reperfusion injury (IRI) on the kidney are ongoing. Recently, there has been an increasing interest in using natural phytochemical compounds as alternative remedies in several diseases. Dihydromyricetin is a flavonoid that is mainly extracted from some plants such as Ampelopsis grossedentata. The effect of dihydromyricetin was investigated in a rat model of renal IRI. Dihydromyricetin was dissolved in a vehicle and administered orally as a single daily dose of 400 mg/kg for 10 days prior to IRI and continued for 3 days after IRI. G‐Sham (*n* = 10) underwent sham surgery, whereas G‐IRI (*n* = 10) and G‐IRI/DHM (*n* = 10) underwent bilateral warm renal ischemia for 35 min and received the vehicle or dihydromyricetin, respectively. Renal functions and histological changes were assessed before starting the medication, just prior to IRI, and 3 days after IRI. Dihydromyricetin significantly attenuated the alterations in serum creatinine and urea, creatinine clearance, urinary albumin, and urinary albumin creatinine ratio. Dihydromyricetin has also significantly mitigated the alterations in renal injury markers, pro‐inflammatory, pro‐fibrotic, and apoptotic cytokines, oxidative stress markers, and histological changes. We conclude that dihydromyricetin has a reno‐protective effect on the IRI‐induced renal alterations. These findings might have clinical implications.

## 1 INTRODUCTION

Renal ischemia–reperfusion injury (IRI) is one of the causes of acute kidney injury and occurs during many clinical procedures and conditions such as renal transplantation, partial nephrectomy, and recovery following systemic hypotension (Weight et al., 1996). It leads to several renal alterations, including a reduction in the glomerular filtration rate (GFR) (Myers et al., 1984). The efforts to reduce the impact of IRI on kidney functions are ongoing (Goes et al., 1995; Hammad, Davis, et al., 2001; Hammad, Wheatley, & Davis, 2001; Petersen & Mitchell, 1981; Troppmann et al., 1995; Unal et al., 2001; Yang et al., 2001).

Dihydromyricetin (DHM), also known as ampeloptin, is a flavonoid that is mainly extracted from the stems and leaves of the Ampelopsis grossedentata, a wild plant that is traditionally used in some countries as a tea and as a treatment for fever and cough (Liu et al., 2019). Like many other flavonoids, DHM was shown to have several biological properties including anti‐inflammatory (Hou et al., 2015; Sun et al., 2021), antioxidant (Li et al., 2021), anti‐cancer (Wu et al., 2022) and antiviral (Xiao et al., 2021) effects with excellent safety and almost no toxicity (Semwal et al., 2016). For instance, DHM has been shown to improve insulin resistance in diabetic rats (Liu et al., 2017). In the mice, DHM prevented non‐alcoholic fatty liver disease by improving mitochondrial respiratory capacity and redox homeostasis in hepatocytes through a SIRT3‐dependent mechanism (Zeng et al., 2019). DHM has also been shown to be protective in Alzheimer's disease in a rat model (Sun et al., 2019). Moreover, it has an anti‐cancer effect in several malignancies including lung (Fan et al., 2017), gastric (Ji et al., 2015) and liver (Jiang et al., 2015) cancer.

In the kidney, few studies have shown DHM to be protective in some conditions. DHM had an inhibitory effect on the formation of vascular calcification in chronic kidney disease (Feng et al., 2021). It has also been shown to promote autophagy and attenuate renal interstitial fibrosis in diabetic nephropathy (Guo et al., 2020). Moreover, its renal protective effects have been demonstrated in diabetes mellitus (Liu et al., 2016), septic acute kidney injury (Tian et al., 2021) and lipopolysaccharide‐induced acute renal injury (Wang et al., 2016). However, the effect of dihydromyricetin on the renal dysfunction following IRI has not been studied yet, and hence this study aimed to investigate this effect in a rat model of bilateral warm renal IRI.

## 2 MATERIALS AND METHODS

Studies were performed on male Wistar rats weighing around 200 g at the time of IRI. Rats were housed in standard rat cages except when daily urine collection is required when they were placed individually in metabolic cages (*vide infra*). They were fed a standard rat chow (National feed and flour Co., Abu Dhabi, UAE). Animals were fasted for 12 h before the experimental procedures but had water *ad libitum*. The experimental protocol was approved by the local ethics committee (ERA‐2023‐2735).

### 2.1 Ischemia–reperfusion injury

All surgical procedures were carried out similar to what has been previously described (Hammad et al., 2021). So, rats were anesthetized by intraperitoneal injection of ketamine (50 mg/kg) (Sigma Aldrich, St Louis, Missouri, USA) and pentobarbitone (30 mg/kg) (Sigma Aldrich). Through a midline abdominal incision, the left and right renal arteries were exposed and clamped simultaneously for 35 min using microsurgical non‐traumatic bulldog clamps. This was followed by re‐perfusion by releasing the arterial clamps. The surgical incision was finally closed in layers using 4/0 vicryl.

### 2.2 Experimental protocol and dihydromyricetin and vehicle administration

**FIGURE 1:**
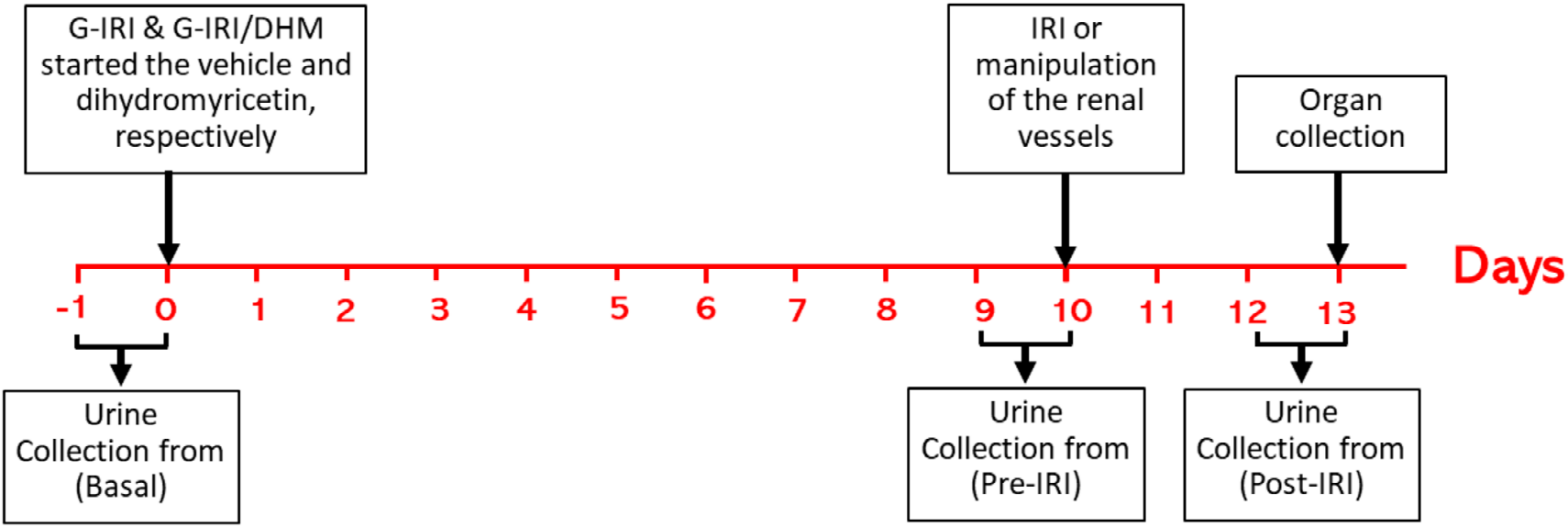
Schematic presentation of the study plan.

Dihydromyricetin (MuseChem, Fairfield, New Jersey, USA) was dissolved in 0.5 mL of the vehicle which is composed of carboxymethyl cellulose 0.5% (Sigma Aldrich), Tween‐80 1.0% (Sigma Aldrich) and polyethylene glycol‐400 1.0% (Sigma Aldrich) in distilled water and administered by oral gavage immediately after preparation as a single daily dose of 400 mg/kg. The dose was similar to the one used by other studies in rats (Liu et al., 2016; Wu et al., 2016). As shown in Figure 1, the treatment started 10 days before the renal IRI surgery and continued for 3 days after the procedure. None of the treated animals showed any adverse effect.

### 2.3 Experimental groups

The rats were assigned randomly to three groups:

- G‐Sham (*n* = 10): Rats which underwent sham manipulation of both renal arteries and received the vehicle.
- G‐IRI (*n* = 10): Rats that underwent warm bilateral renal ischemia for 3**5** min and received only the vehicle.
- G‐IRI/DHM (*n* = 10): Rats which underwent bilateral renal ischemia for 35 minutes and received dihydromyricetin dissolved in the vehicle.

### 2.4 Sample collection and analysis

Urine was collected using metabolic cages for 24 h at three different time points: just before the start of dihydromyricetin/vehicle treatment for baseline pre‐treatment values (Basal), day 9 after dihydromyricetin/vehicle treatment for pre‐IRI values (Pre‐IRI) and on the third day after IRI (Post‐IRI). The volume of daily urine at these time points were calculated (Figure 1). Using the tail vein, blood was withdrawn at the same day of urine collection. All samples were frozen at −30°C for later measurement of urea, albumin and creatinine levels. Seventy‐two hours post‐IRI, the animals were anesthetized using intraperitoneal injection of pentobarbitone (50 mg/kg) and the kidneys were collected and stored in either liquid nitrogen then at −80°C or in formalin for further assays. The animals were euthanized by removal of the heart under anesthesia after obtaining the required tissues.

### 2.5 Gene expression analysis

A wedge of left renal tissues containing both cortex and medulla was excised. It was then snap‐frozen in liquid nitrogen and stored at −80°C for later measurement of gene expression, using r*everse* transcription *polymerase chain reaction* (RT‐PCR), of the following cytokines and markers:

1. Acute kidney injury markers: Kidney injury molecule‐1 (KIM1) and neutrophil gelatinase‐associated lipocalin (NGAL).
2. Cytokines involved in the inflammation and fibrosis: *Tumor necrosis factor‐α* (TNFα), transforming growth factor‐β (TGF‐β1), and monocyte chemoattractant protein‐1 (MCP‐1).
3. The pro‐apoptotic gene p53.
4. The antioxidant enzymes, glutathione peroxidase (GPX‐1), and glutathione‐disulfide reductase (GSR).

Extraction of the total RNA from the frozen samples was performed using Qiazol Lysis reagent (Qiagen, Hilden, Germany) as per the manufacturer's protocol. Estimation of the quantity and quality of the extracted RNA was performed using NanoDrop 2000 Spectrophotometer (Thermo Fisher Scientific Inc., Wilmington, Delaware, USA).

Preparation of the complementary DNA (cDNA) in duplicates from 1.0 μg of extracted RNA was achieved using the FIREScript® RT cDNA synthesis kit (Solis BioDyne, Tartu, Estonia) as per the manufacturer's protocol. Subsequently, the prepared cDNA was used as a template for relative gene expression analysis using hydrolysis probe chemistry. The reaction mixture contained 50 ng cDNA, GoTaq® Probe qPCR master mix (Promega Corporation, Madison, *Wisconsin*, USA), 0.5 μM of forward and reverse primers, and 0.25 μM of fluorescent probe (Biosearch Technologies Inc., Petaluma, California, USA). The probes were FAM‐labeled.

Peptidylprolyl Isomerase A (PPIA) housekeeping gene (Promega Corporation) was used for normalization. Its probe was labeled with Quasar 670 (Promega Corporation) enabling the multiplexing with the genes of interest. All samples were run in duplicates. At least one primer of all designed PCR primer sets was spanning the exon‐exon junction to further exclude any interference from the genomic DNA. Table 1 shows the sequences of primers and probes (Promega Corporation). The calculated Cycle Threshold (CT) values were used in the estimation of the changes in gene expression of target genes using the delta–delta CT formula.

**TABLE 1:** Forward and reverse primers and fluorogenic probe sequences used for real‐time quantitative PCR analysis.

KIM‐1 (NM_173149.2)	Forward	GCCTGGAATAATCACACTGTAAG
	Reverse	GCAACGGACATGCCAACATAG
	Probe	d FAM‐TCCCTTTGAGGAAGCCGCAGA‐BHQ‐1
Lipocalin 2 (LCN2) (NM_130741.1)	Forward	CTGTTCCCACCGACCAATGC
	Reverse	CCACTGCACATCCCAGTCA
	Probe	FAM‐TGACAACTGAACAGACGGTGAGCG‐BHQ‐1
TNF‐α (NM_012675.3)	Forward	CTCACACTCAGATCATCTTCTC
	Reverse	CCGCTTGGTGGTTTGCTAC
	Probe	FAM‐CTCGAGTGACAAGCCCGTAGCC‐BHQ‐1
TGF‐β1 NM_012620.1	Forward	GTGGCTGAACCAAGGAGACG
	Reverse	CGTGGAGTACATTATCTTTGCTGTC
	Probe	FAM‐ACAGGGCTTTCGCTTCAGTGCTC‐BHQ‐1
MCP‐1	Forward	GCTGTCTCAGCCAGATGCAG
	Reverse	CCAGCCGACTCATTGGGA
	Probe	FAM‐CCCACTCACCTGCTGCTACTCA‐BHQ‐1
p53 (NM_030989.3)	Forward	CGAGATGTTCCGAGAGCTGAATG
	Reverse	GTCTTCGGGTAGCTGGAGTG
	Probe	FAM‐CCTTGGAATTAAAGGATGCCCGTGC‐BHQ‐1
PPIA (NM_017101.1)	Forward	GCGTCTGCTTCGAGCTGT
	Reverse	CACCCTGGCACATGAATCC
	Probe	Quasar 670‐TGCAGACAAAGTTCCAAAGACAGCA‐BHQ‐2

Abbreviations: KIM‐1, Kidney injury molecule‐1; NGAL, Neutrophil gelatinase‐associated lipocalin also called lipocalin 2 (Lcn2); p53, Pro‐apoptotic gene p53; PPIA, Peptidylprolyl isomerase A (housekeeping gene); TGF‐β1, Transforming growth factor‐β; TNF‐α, Tumor necrosis factor‐alpha.

The results were expressed as the mean fold change of gene expression compared to the G‐Sham. The gene expression of the G‐Sham animals was measured and the average was calculated. This average was given a value of 1. To this value, all other groups including the animals of the G‐Sham were compared.

### 2.6 Western blot analysis

Proteins were extracted from frozen kidney samples using freshly made 1X RIPA buffer (0.05 M Tris–HCl (pH 7.4), 0.15 M NaCl, 1 mM Na_2_EDTA, 1% Igepal, 0.25% sodium deoxycholate, 1 μg/mL proteinase inhibitor cocktail (Thermo Fisher Scientific Inc.)). Equal amounts of extracted proteins (15–30 g) were diluted in 5X loading buffer and loaded onto 12% SDS‐PAGE gels (Thermo Fisher Scientific Inc). The proteins were transferred onto a polyvinylidene difluoride (PVDF) membrane and then blocked with 5% non‐fat milk diluted in PBS‐T (0.5% Tween‐20). The membranes were incubated with the primary antibody overnight at 4°C. The primary antibodies used were as follows: anti‐p‐NF‐κB p65 (1:1000, CAT# 3033S, Cell Signaling Technology® (CST), Danvers, Massachusetts, USA), anti‐cleaved caspase‐3 (1:1000, CAT# 9664S, CST), anti‐IL‐1β (1/5000, CAT# 10663‐1‐AP, Protein Tech), and GAPDH (1:1000, CAT # 2118S, CST). All blots were then incubated at room temperature with diluted goat anti‐rabbit secondary antibodies conjugated with horseradish peroxidase enzyme (1:20000) (Abcam, Cambridge, UK). The bands were visualized using a chemiluminescence West Pico kit, and signals were detected using X‐ray film. Quantification of the bands was performed using ImageJ software (NIH, Milwaukee, WI, USA). Densitometry analysis of the detected bands was done using the NIH ImageJ analysis software (National Institute of Health, Stapleton, New York, USA).

### 2.7 Histological studies

The kidney tissue was washed with ice‐cold saline and blotted using filter paper, cassetted and fixed directly in 10% neutral formalin for 24 h. This was followed by dehydration in increasing ethanol concentrations, clearing with xylene, and embedding with paraffin. Four‐μm sections were prepared from paraffin blocks and stained with hematoxylin and eosin. The stained sections were evaluated blindly using light microscopy.

The pathological scoring of the kidney sections depended on the extent of the field which showed cortical and medullary tubular injury. A scale of 0–4 was used (0, no tubular injury; 1, tubular injury presents in ≤25% of the fields; 2 tubular injury presents in 26%–50% of the fields; 3, tubular injury presents in 51%–75% of the fields; 4, tubular injury presents in 76%–100% of the fields). Image J software (NIH) was used to measure the extent of necrosis.

### 2.8 Statistical analysis

Statistical analysis was performed using SPSS V16.0. Results were expressed as mean ± SD. One‐way factorial ANOVA was used for comparison of variables between groups and between different stages (Basal, Pre‐IRI, and Post‐IRI) within each group. *p* value less than 0.05 was considered statistically significant.

## 3 RESULTS

As demonstrated in Table 2 and Table 3, the serum creatinine, serum urea, creatinine clearance, 24‐h urinary albumin and albumin/creatinine ratio before starting the medication/vehicle ‘Basal’, were similar in all the groups (*p* > 0.05 for all variables). Likewise, the Basal and Pre‐IRI values in all the groups were not different (*p* > 0.05 for all variables).

**TABLE 2:** Serum creatinine, serum urea, and creatinine clearance in all the groups at different stages.

	Group		
	G‐Sham	G‐IRI	G‐IRI/DHM
*S. creatinine* (mg/dL)			
Basal	0.21 ± 0.02	0.21 ± 0.03	0.19 ± 0.03
Pre‐IRI	0.20 ± 0.02	0.19 ± 0.03	0.22 ± 0.02
Post‐IRI	0.19 ± 0.02	0.80 ± 0.69^a^	0.34 ± 0.12^a^, ^b^
*S. urea* (mg/dL)			
Basal	48.1 ± 2.8	49.0 ± 4.8	50.3 ± 8.4
Pre‐IRI	49.2 ± 5.3	50.9 ± 7.4	48.4 ± 3.6
Post‐IRI	47.3 ± 2.0	151.7 ± 88.1^a^	78.8 ± 23.0^a^, ^b^
Creatinine clearance (mL/min/100 g)			
Basal	1.05 ± 0.28	1.03 ± 0.16	1.04 ± 012
Pre‐IRI	1.02 ± 0.04	1.08 ± 0.20	0.96 ± 0.12
Post‐IRI	1.06 ± 0.32	0.37 ± 0.19^a^	0.75 ± 0.20^a^, ^b^

*Note*: Basal: Before the administration of dihydromyricetin/vehicle; Pre‐IRI: After the administration of the dihydromyricetin/vehicle and just before the ischemia–reperfusion injury; Post‐IRI: And on the third day following IRI. Values are expressed as mean ± SD.

^a^ Statistical significance when compared to the Pre‐IRI within the same group;

^b^ Statistical significance when compared to the G‐IRI group.

**TABLE 3:** 24‐h urinary albumin and albumin/creatinine ratio in all the groups at different stages.

	Group		
	G‐Sham	G‐IRI	G‐IRI/NR
24‐h Urinary Albumin (μg)			
Basal	6.46 ± 3.92	6.54 ± 1.53	6.41 ± 1.99
Pre‐IRI	5.57 ± 3.85	5.66 ± 3.71	5.32 ± 3.17
Post‐IRI	5.83 ± 2.90	124.83 ± 30.29^a^	87.22 ± 20.28^a^, ^b^
Albumin/Creatinine Ratio			
Basal	13.5 ± 1.4	11.1 ± 3.6	12.6 ± 5.4
Pre‐IRI	11.4 ± 5.0	12.3 ± 3.2	11.0 ± 4.8
Post‐IRI	14.4 ± 1.7	199.1 ± 30.4^a^	127.0 ± 39.2^a^, ^b^

*Note*: Basal: Before the administration of dihydromyricetin/vehicle; Pre‐IRI: After the administration of the dihydromyricetin/vehicle and just before the ischemia–reperfusion injury; Post‐IRI: And on the third day following IRI. Values are expressed as mean ± SD.

^a^ Statistical significance when compared to the Pre‐IRI within the same group.

^b^ Statistical significance when compared to the G‐IRI group.

As expected, in the G‐Sham, the post‐manipulation values of all variables were similar to the Pre‐IRI values (*p* > 0.05 for all variables). In the G‐IRI, there was a significant deterioration in renal functions after IRI (Table 2). For example, there was a significant increase in serum creatinine from 0.19 ± 0.02 to 0.80 ± 0.69 mg/dL (*p* < 0.05) and creatinine clearance has decreased to 0.37 ± 0.19 compared to 1.06 ± 0.32 mL/min/100 g b.w. before IRI (*p* < 0.01). Further, IRI has caused an increase in the urinary albumin leak. So, the 24‐h urinary albumin and albumin/creatinine ratio have increased to 124.83 ± 30.29 μg (vs. 5.83 ± 2.90) and 199.1 ± 30.4 (vs. 14.4 ± 1.7) (*p* < 0–0001 for both), respectively (Table 3).

As shown in Tables 2 and 3, the administration of dihydromyricetin has significantly attenuated the IRI‐induced alterations in these parameters (*p* < 0.05 for all variables).

### 3.1 Gene expression analysis results

**FIGURE 2:**
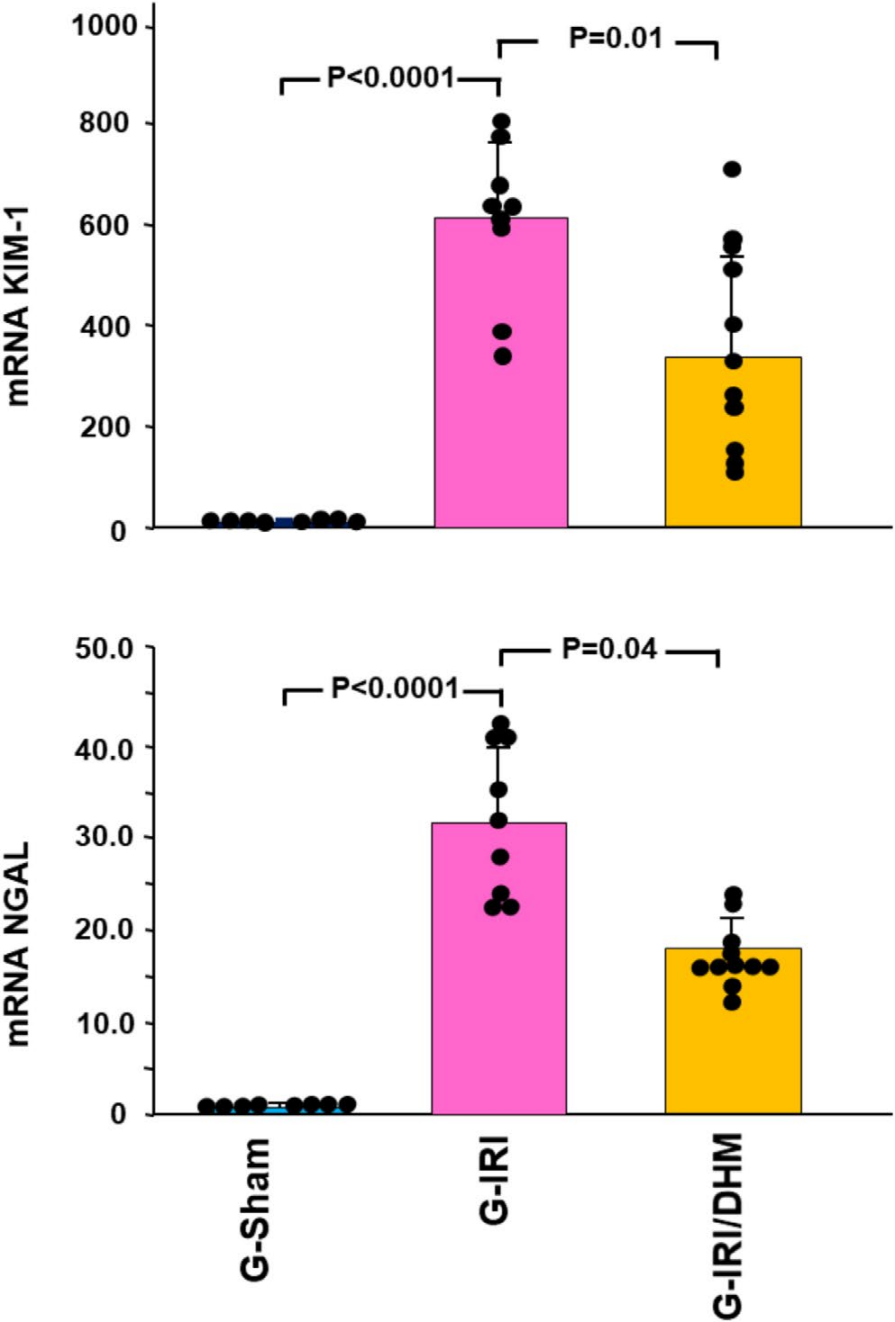
The gene expression of two of markers of acute renal injury (kidney injury molecule‐1 (KIM1) and neutrophil gelatinase‐associated lipocalin (NGAL) in all the groups 3 days following the IRI/manipulation. Values represent mean ± SD.

IRI caused a significant increase in the gene expression of KIM‐1 and NGAL when measured 3 days following IRI (622.5 ± 148.4 vs. 1.1 ± 0.5, and 32.3 ± 8.1 vs. 1.0 ± 0.2, respectively, *p* < 0.0001 for both) (Figure 2). Dihydromyricetin administration has significantly attenuated this gene expression increase in KIM‐1 and NGAL (370.0 ± 201.3 vs. 622.5 ± 148.4, *p* < 0.01 and 17.0 ± 3.3 vs. 32.3 ± 8.1, *p* < 0.05, respectively).

**FIGURE 3:**
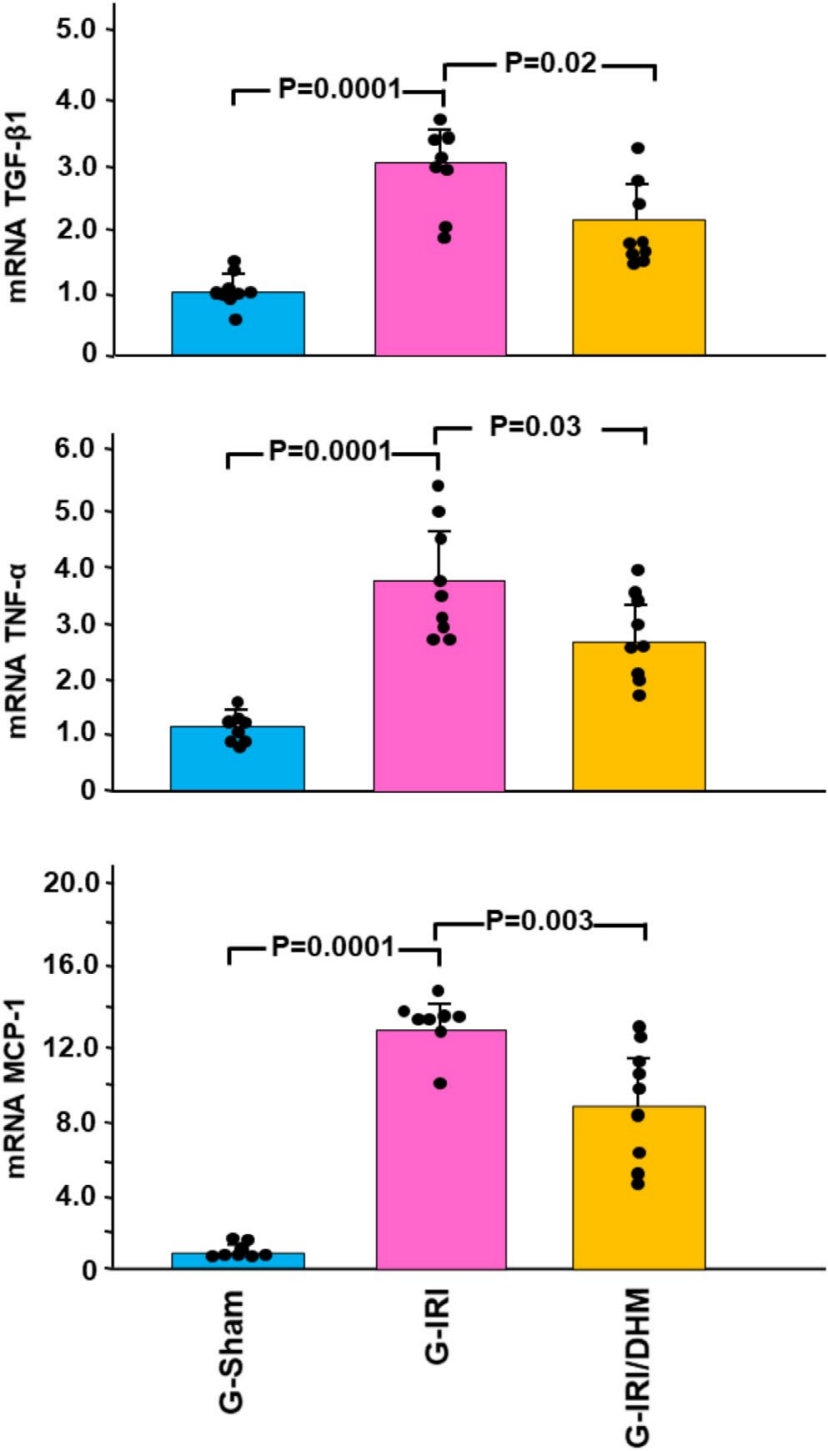
The gene expression of the cytokines involved in the inflammation and fibrosis: *Tumor necrosis factor‐α* (TNFα), transforming growth factor‐β (TGF‐β1), and monocyte chemoattractant protein‐1 (MCP‐1) in all groups 3 days following the IRI/manipulation. Values represent mean ± SD.

**FIGURE 4:**
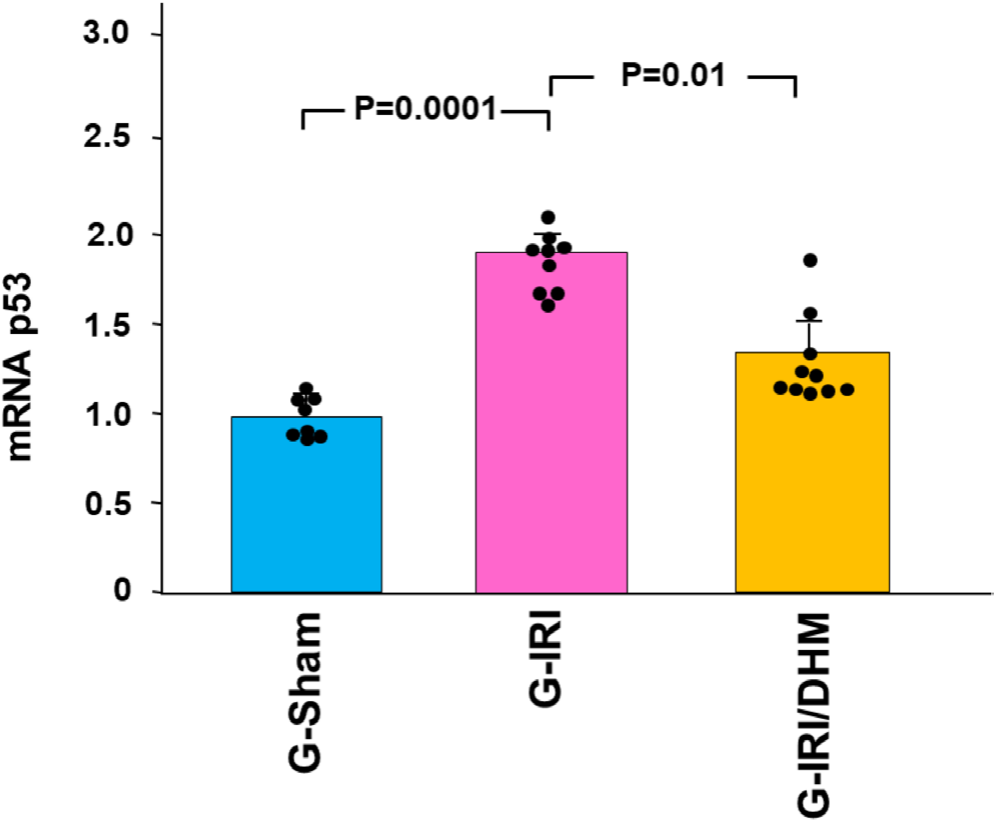
The gene expression of the pro‐apoptotic p53 gene in all groups 3 days following the IRI/manipulation. Values represent mean ± SD.

Likewise, a similar trend was noticed with pro‐inflammatory, pro‐fibrotic, and pro‐apoptotic cytokines (Figures 3 and 4). For instance, IRI caused a significant increase in the gene expression of TGF‐β1 in the G‐IRI compared to G‐Sham (3.01 ± 0.60 vs. 1.04 ± 0.28, *p* < 0.001). Dihydromyricetin significantly attenuated this increase (2.19 ± 0.67 vs. 3.01 ± 0.60, *p* < 0.05). Similar effects were observed with TNF‐α, MCP‐1, and p53 (*p* < 0.05 for all these mediators).

**FIGURE 5:**
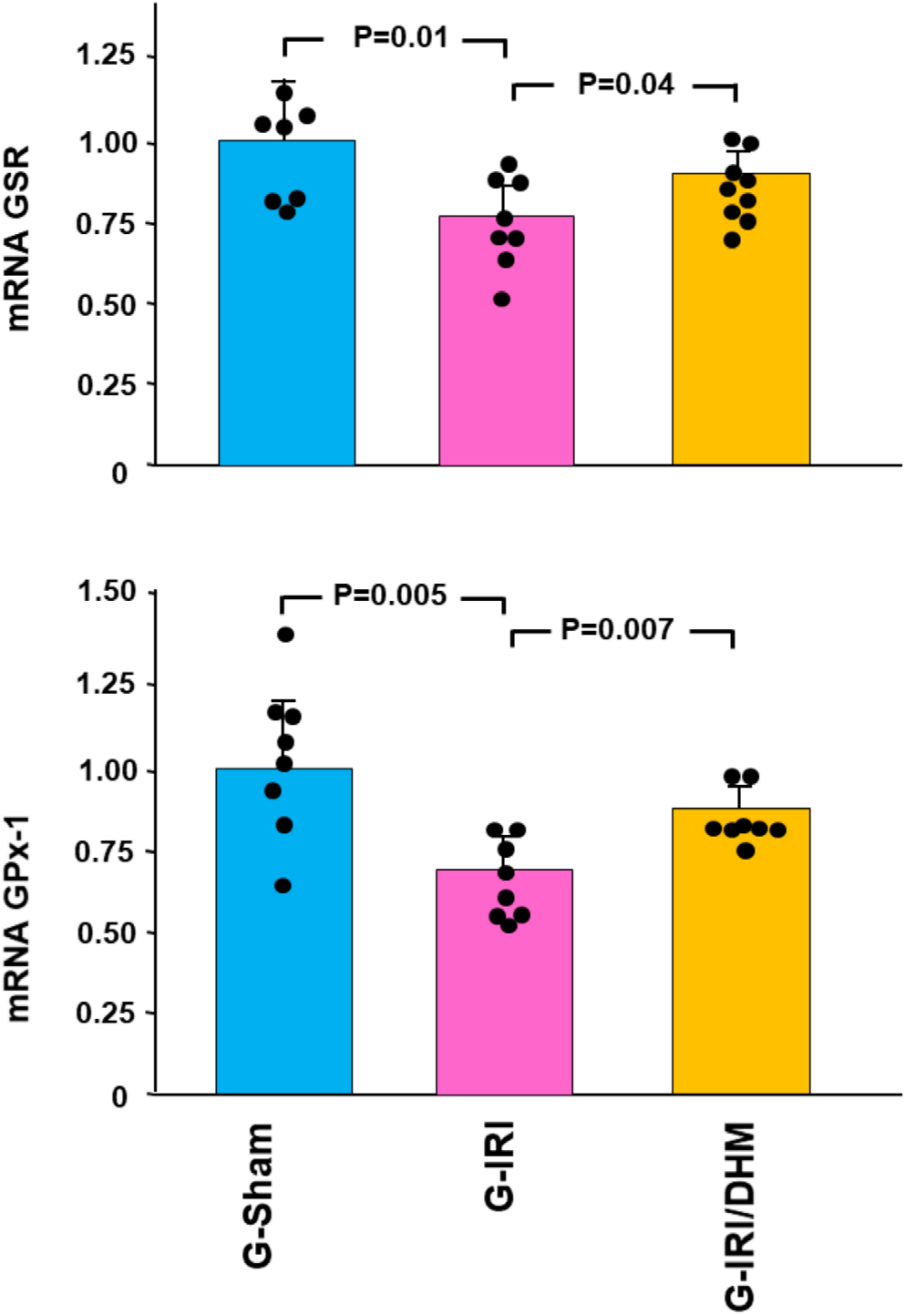
The gene expression of the antioxidant enzymes, glutathione peroxidase (GPX‐1), and glutathione‐disulfide reductase (GSR) in all groups 3 days following the IRI/manipulation. Values represent mean ± SD.

Dihydromyricetin has also significantly attenuated the change in the gene expression of the oxidative stress markers GPX‐1 and GSR (Figure 5). For instance, IRI led to a significant decrease in the gene expression of GSR (0.76 ± 0.11 vs. 1.02 ± 0.21, *p* < 0.05) and this was attenuated by dihydromyricetin (0.90 ± 0.12 vs. 0.76 ± 0.11, *p* < 0.05).

### 3.2 Western blot analysis

**FIGURE 6:**
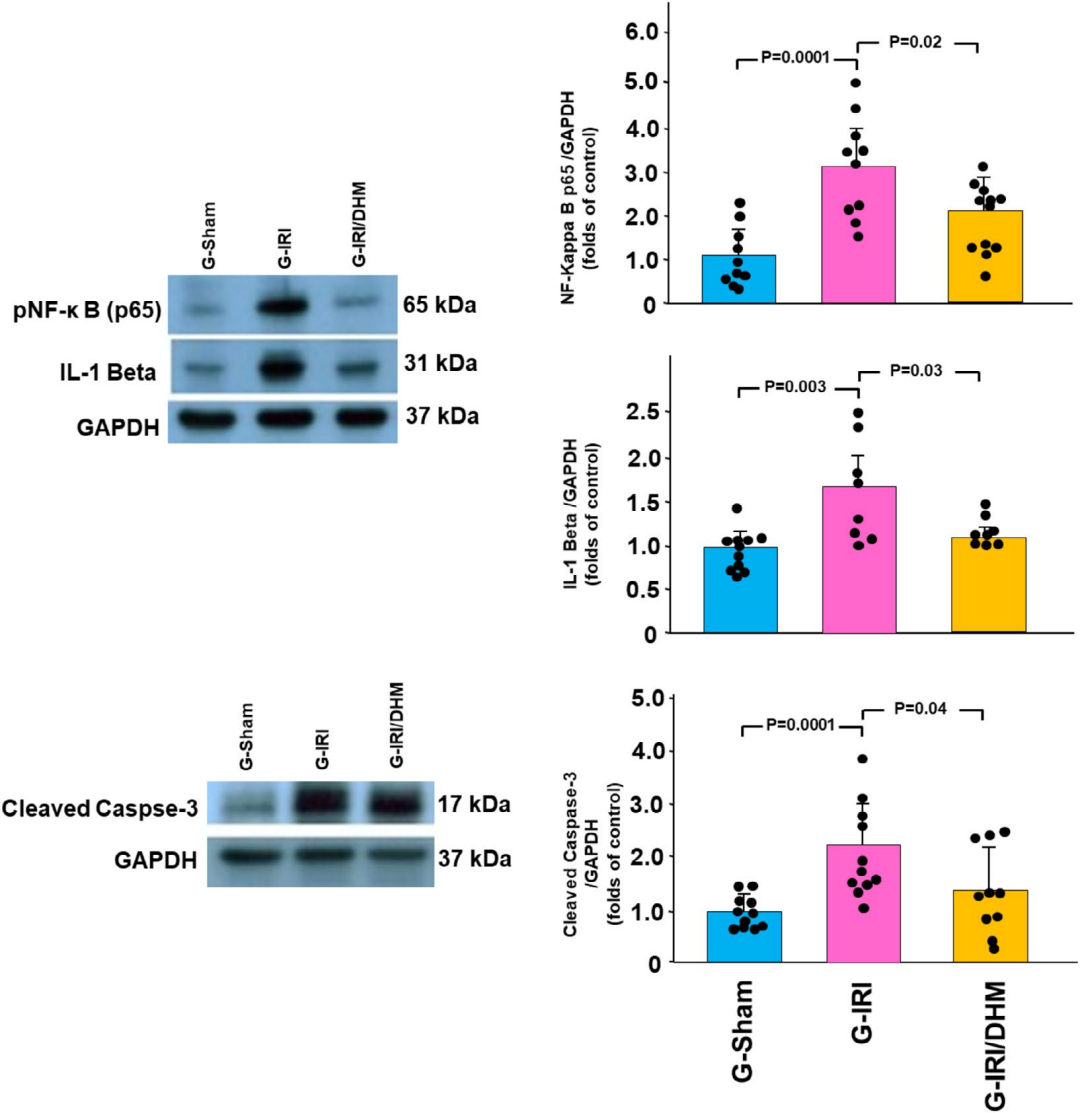
The protein expression of pNF‐κB p65, IL‐1β, and cleaved caspase‐3 (normalized to GAPDH protein expression) in all groups 3 days following the IRI/manipulation. Values are expressed as the ratio of mean ± SD relative to the Sham group.

As shown in Figure 6, NF‐Kappa B p65 tissue concentration was significantly higher in the G‐IRI compared to the G‐Sham (3.11 ± 1.22 vs. 1.00 ± 0.79, *p* < 0.001) when measured 3 days following IRI. Dihydromyricetin attenuated this rise (1.99 ± 0.85 vs. 3.11 ± 1.22, *p* < 0.05). Similar findings were obtained in cleaved caspase‐3 and IL‐1β.

### 3.3 Histological studies

**FIGURE 7:**
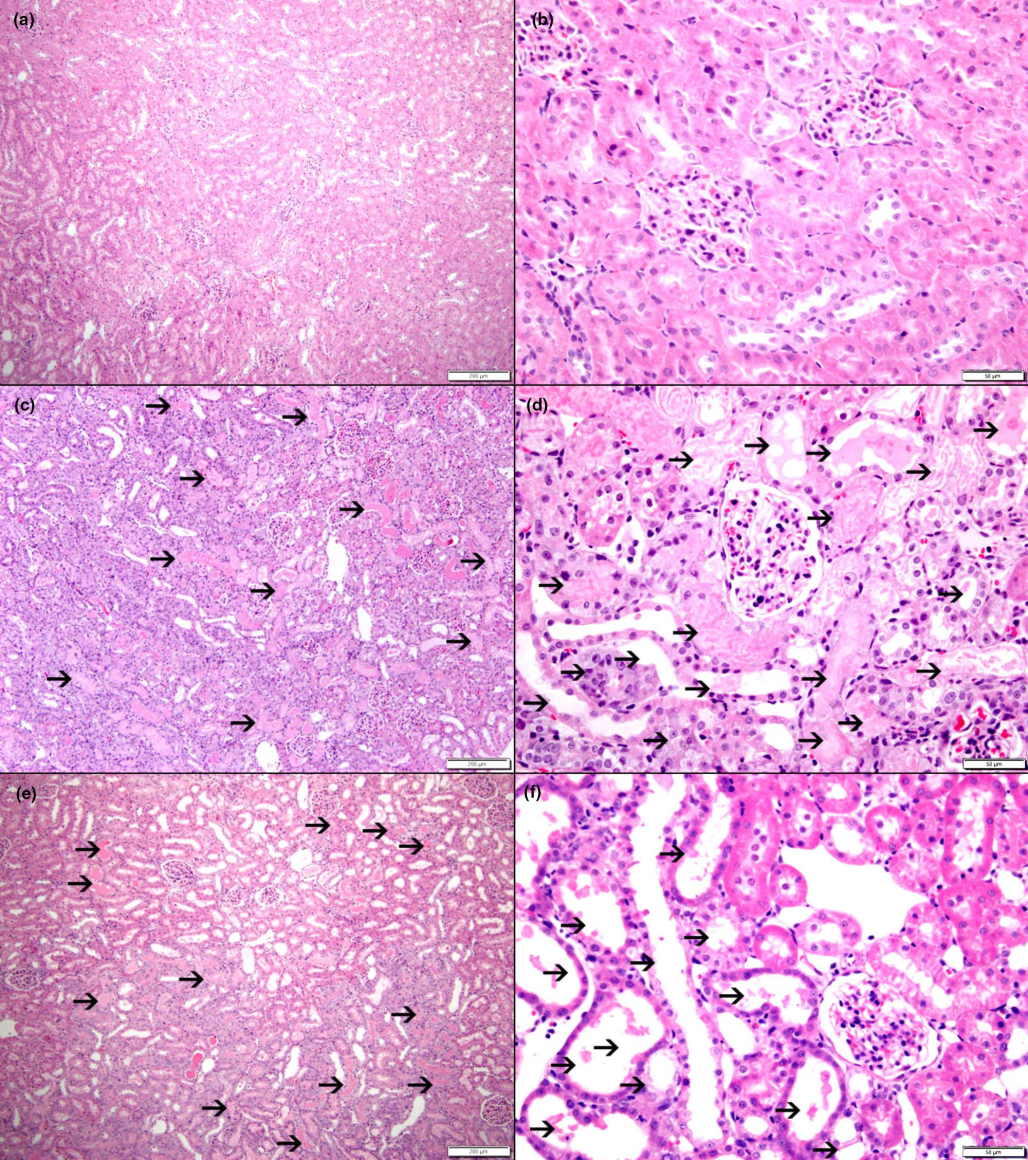
The histological features in all the groups. (a, b) The kidneys in G‐Sham showing normal histological features. (c, d) represent the histological features in the G‐IRI showing large areas of acute tubular necrosis with tubular dilatation and intratubular secretions (thin arrows) These abnormalities occupied 91.2 ± 3.4% of the field compared to 0% in the G‐Sham group (*p* < 0.0001). (e, f) show the kidney tissue in G‐IRI/DHM with a milder degree of acute tubular necrosis including tubular dilatation and intratubular secretions which occupied 79.3 ± 5.5% of the fields (*p* = 0.02 when compared to G‐IRI) (thin arrows).

As shown in Figure 7a,b, the G‐Sham showed normal kidney architecture and histology. The G‐IRI had large areas of acute tubular injury with dilated renal tubules and intratubular eosinophilic secretion, which occupied 91.2 ± 3.4% of the examined fields (*p* < 0.0001 when compared to G‐Sham) (Figure 7c,d). As shown in Figure 7e,f, dihydromyricetin attenuated the effect of IRI, as the G‐IRI/DHM kidney had only 79.3 ± 5.5% of the fields showing acute tubular injury (*p* = 0.02 when compared to G‐IRI).

## 4 DISCUSSION

In the current study, we have investigated, for the first time, the effect of dihydromyricetin on the renal dysfunction caused by IRI. We have shown that the administration of this natural product prior to and after IRI has significantly attenuated the IRI‐induced alterations in the renal functional parameters such as serum creatinine, creatinine clearance and urinary albumin leakage. It has also significantly mitigated the changes in the gene expression or the tissue level of acute renal injury markers, pro‐inflammatory, pro‐fibrotic, and pro‐apoptotic cytokines and of oxidative stress markers. Our data has also shown a significant improvement in the histopathological abnormalities.

The IRI‐induced renal damage is caused by both ischemia and reperfusion which lead to hypoxia and excessive production of reactive oxygen species. This in turn initiates an inflammatory response characterized by increased production of several cytokines such as pro‐inflammatory and pro‐fibrotic cytokines including TNF‐α and TGF‐β1 (Chatauret et al., 2014; Furuichi et al., 2008; Hammad et al., 2012; Hammad et al., 2013; Lerman & Textor, 2001; Spurgeon et al., 2005). The inflammatory changes with the subsequent fibrosis determine the extent of the renal damage.

In the current study, dihydromyricetin had a significant antioxidant effect as demonstrated by its effects on the gene expression of some of the oxidative stress enzymes namely glutathione peroxidase and glutathione‐disulfide reductase. The antioxidant and free radical scavenging properties of dihydromyricetin has been demonstrated in other injury models (Du et al., 2024; Li et al., 2017; Li et al., 2021). The contradictory down‐regulation in the gene expression of the oxidative stress enzymes associated with IRI demonstrated in this study has been previously observed in other studies in this condition (Dobashi et al., 2000; Jiang et al., 2021) and other conditions (AlAsmari et al., 2022; Hammad et al., 2019) in which the oxidative stress plays a major role in the pathophysiology of the disease process. This down‐regulation has been attributed to the higher turnover of these enzymes due to excessive production of reactive oxygen species (Dobashi et al., 2000). Regardless of the exact explanation, the administration of dihydromyricetin in the current study had mitigated this alteration in oxidative stress enzymes.

In addition to its antioxidant properties, the current study demonstrated an anti‐inflammatory and anti‐fibrotic effect of dihydromyricetin as shown by the attenuation in IRI‐induced rise in the gene expression of TNF‐α, TGF‐β1, and MCP‐1. TNF‐α is a pro‐inflammatory cytokine which is produced by several tissues including renal cells (Speeckaert et al., 2012). MCP‐1 plays a role in the chemoattraction of macrophages and T‐cells to the injury site (Deshmane et al., 2009; Sung et al., 2002). TGF‐β is a pro‐fibrotic cytokine that stimulates renal cells to produce extracellular matrix proteins which lead to long‐term glomerulosclerosis and tubulointerstitial fibrosis (Ding & Choi, 2014; Spurgeon et al., 2005). The mitigation of the alterations in all these important cytokines by dihydromyricetin indicates a significant anti‐inflammatory and anti‐fibrotic effects of this natural product in IRI.

This anti‐inflammatory effect of dihydromyricetin was further demonstrated by the attenuation of the alterations of the tissue level of pNF‐κB p65 as shown by the western blot assay. In the resting sate, NF‐kB is found in the cytosol in an inactive form due to IκB inhibitory proteins (Giridharan & Srinivasan, 2018). The activation of NF‐κB depends on the phosphorylation‐induced ubiquitination of IκB proteins and leads to its nuclear translocation and the transcriptional activation of pro‐inflammatory cytokines such as IL‐1β, TNF‐α, and MCP‐1 (Sung et al., 2002). The gene expression or the protein level of all such cytokines were shown to be altered by IRI in this study and the administration of dihydromyricetin had ameliorated such alterations. Therefore, it is reasonable to presume that the protective effect of dihydromyricetin on the pro‐inflammatory cytokine alterations might be, at least partially, through its effects on the expression of NF‐κB.

In addition to its anti‐inflammatory and antioxidant properties, dihydromyricetin was shown to have anti‐apoptotic properties as demonstrated by the mitigation of the alterations in the gene expression of p53 and protein expression of cleaved caspase‐3. From the current data, it is difficult to ascertain if dihydromyricetin had affected other apoptotic pathways or apoptotic markers which were shown to be activated in renal IRI such as cytochrome‐c (Li et al., 2024) and Fas‐ligand (Zhu et al., 2019) and further studies are required to address this point. This p53/caspase‐3 dependent anti‐apoptotic effect which was demonstrated in this study, in addition to the anti‐inflammatory and antioxidant properties of the dihydromyricetin led to the improvement of renal functions such as serum creatinine and creatinine clearance in comparison to the non‐treated group. The improvement in urinary albumin leak in addition to these parameters indicated that this natural product does not only affect the glomerular function but the renal tubular function as well. This was supported by its significant effects on the gene expression of some of the acute renal injury markers such as NGAL and KIM‐1. The latter is strongly expressed and released by the injured proximal renal tubular cells (Lim et al., 2013) whereas NGAL is synthesized in the distal segments such as the thick ascending limb of Henle's loop and collecting ducts (Mishra et al., 2003). Therefore, the current data indicates that dihydromyricetin has an effect on different segments of renal tubules.

From the current data, it is difficult to ascertain the exact pharmacological mechanism through which dihydromyricetin led to these reno‐protective effects. One possible mechanism is the dihydromyricetin's powerful scavenging properties of the harmful free radicals similar to other flavonoids (Burda & Oleszek, 2001). This is possibly due to the free hydroxyl in the C‐3 position in C‐ring and o‐dihydroxy system (hydroxyl in the C‐3′ and 4’ position) in the B‐ring, which has been shown to have strong effect against free radicals (Burda & Oleszek, 2001). The anti‐inflammatory effects of flavonoids are also shown to be closely linked to their antioxidant effects (Shoham et al., 2008). In this regard, reactive oxygen species has not been demonstrated to exist only in oxidation process but also to be involved in regulating the expression of target genes related to inflammation such as NF‐κB (Hoffmann & Baltimore, 2006). Regardless of the exact pharmacological mechanism, dihydromyricetin appears to have powerful protective effects in renal IRI.

The model used in the current study is similar to the clinical scenario of the renal ischemia encountered in some surgical procedures such as renal transplantation and partial nephrectomy. These procedures are performed worldwide with an increasing rate and hence, the protective effects of dihydromyricetin which was demonstrated in the current study might be clinically relevant in such a way that these patients might benefit from taking this agent in the peri‐operative period. However, further clinical research is required to extrapolate these results to the clinical set‐up.

In conclusion, the administration of dihydromyricetin before and after renal IRI have ameliorated the IRI effects on the renal functional parameters, alterations in markers of kidney injury and histological features. The current data indicates that the protective effects are due to its anti‐inflammatory, anti‐fibrotic, anti‐apoptotic and anti‐oxidative properties. These findings might have clinical implications.

## AUTHOR CONTRIBUTIONS

Conceptualization, F.T.H. and L.L.; Methodology, L.L., M.A., A.A.A., A.F.H., W.F.H., S.A., M.E.H., F.T.H.; Software, F.T.H., L.L.; Formal analysis, F.T.H., L.L., S.A., J.A.R.; Resources, F.T.H.; Data curation, F.T.H., L.L., M.A. and S.A.; Writing‐original draft, F.T.H.; Writing‐review and editing, F.T.H, L.L., M.A., A.A.A., A.F.H., W.F.H., S.A., M.E.H., J.A.R.; Supervision, F.T.H.; Project administration, F.T.H.; Funding acquisition, F.T.H.

## FUNDING INFORMATION

This research was funded by the College of Medicine and Health Sciences, United Arab Emirates University (SURE+ grant, 2023).

## CONFLICT OF INTEREST STATEMENT

None of the authors has any conflict of interest to declare.

## ETHICS STATEMENT

The study was conducted according to the guidelines of the Declaration of Helsinki and approved by the College of Medicine and Health Sciences, UAEU Animal Ethics Committee (ERA‐2023‐2735).

## Data Availability

The data presented in this study are available on request from the corresponding author.
